# Cesarean Sections Under Spinal Anaesthesia: Comparison of Varying Doses of Dexmedetomidine Combined with 0.75% Hyperbaric Ropivacaine: A Double-Blind Randomized Trial

**DOI:** 10.4274/TJAR.2024.241619

**Published:** 2024-09-17

**Authors:** Srinivasa Rao Nallam, Srikavya Kandala, Sreelekha Kanipakam, Vinay Bathini, Sunil Chiruvella, Sonu Sesham

**Affiliations:** 1 Dr YSR Government Medical College, Department of Anaesthesia, Critical Care & Pain Medicine, Pulivendula, India; 2 Government Medical College, Department of Anaesthesia, Critical Care & Pain Medicine, Kadapa, India

**Keywords:** Analgesia, cesarean section, dexmedetomidine, ropivacaine, spinal anaesthesia

## Abstract

**Objective:**

The primary aim of this study was to evaluate the effects of 5 µg, 7.5 µg, and 10 µg doses of dexmedetomidine added to hyperbaric 0.75% ropivacaine on the duration of analgesia during cesarean section. Furthermore, the onset of sensory and motor block, hemodynamics, sedation, and adverse effects were investigated.

**Methods:**

A total of 120 full-term parturients scheduled for cesarean section under spinal anaesthesia were randomized into three groups. Group RD5 received intrathecal hyperbaric 0.75% ropivacaine 15 mg (2 mL) plus dexmedetomidine 5 µg (0.5 mL), group RD7.5 received intrathecal hyperbaric 0.75% ropivacaine 15 mg (2 mL) plus dexmedetomidine 7.5 µg (0.5 mL), and group RD10 received intrathecal hyperbaric 0.75% ropivacaine 15 mg (2 mL) plus dexmedetomidine 10 µg (0.5 mL). Sensorimotor blockade characteristics, analgesia duration, hemodynamic variables, and adverse events were documented. Student’s t-test and the chi-square test were used for data analysis.

**Results:**

In groups RD5, RD7.5, and RD10, the onset of sensory block was 2.96±1.32 min, 2.26±1.50 min, and 1.96±0.93 min, respectively, while the onset of motor block was 9.63±0.11 min, 8.63±0.58 min, and 6.40±0.14 min, respectively. The duration of analgesia was significantly prolonged in group RD10 compared with groups RD7.5 and RD5 (483.43±76.21 vs. 398.74±73.59 vs. 362.58±79.87 min, respectively, *P*=0.001). Group RD10 also exhibited significantly higher incidences of sedation, bradycardia, and vomiting.

**Conclusion:**

We conclude that increasing dexmedetomidine doses decreases the onset of sensory and motor blockade while prolonging analgesia duration in a dose-dependent manner.

**Main Points**

• Currently, subarachnoid block is the ideal anaesthesia option for lower-segment cesarean section deliveries.

• The main limiting factor of spinal anaesthesia is the relatively short duration of anaesthesia and analgesia, which can be overcome by adding adjuvants to intrathecal ropivacaine.

• Our goal is to ascertain the ideal intrathecal dexmedetomidine dose as an adjuvant to 0.5% hyperbaric ropivacaine for prolonging postoperative analgesia without significant adverse effects in parturients.

## Introduction

The ideal option for cesarean section is spinal anaesthesia, provided that there are no contraindications.^1^ One of the very common adverse effects of spinal anaesthesia is hypotension, which is closely related to maternal and neonatal morbidity and mortality. Numerous studies have indicated that the incidence of spinal-induced hypotension can be reduced by reducing the dosage of intrathecal local anaesthetic agent.^2, 3^ However, this reduction in anaesthesia dosage is associated with shorter anaesthesia and analgesia durations. To overcome these disadvantages, various adjuvants, such as opioids, epinephrine, α2 agonists, etc., are recommended.^4^

In recent times, hyperbaric ropivacaine heavy has gained popularity owing to its lower potential for central nervous and cardiac toxicity compared with bupivacaine heavy. However, ropivacaine exhibits less potency, and the motor block duration is shorter than that of bupivacaine.^5^ As a result, spinal anaesthesia using hyperbaric ropivacaine is primarily reserved for cesarean sections.^6^ Numerous studies have explored the efficacy of intrathecal ropivacaine in combination with adjuvants such as fentanyl and sufentanil for cesarean delivery.^7, 8^

Dexmedetomidine exhibits eightfold higher affinity for alpha 2 receptors in contrast to clonidine, and it is a selective alpha 2 agonist. In clinical practice, dexmedetomidine is widely used as an additive in local, regional, and general anaesthesia. Although dexmedetomidine has been approved by the Food and Drug Administration for intravenous (i.v) sedation in the intensive care unit, it has recently become a popular adjuvant to local anaesthetic agents. When dexmedetomidine is used in combination with local anaesthetics in subarachnoid block, it elongates the timespan of sensory and motor blocks, as well as postoperative analgesia, without causing significant sedation.^9^

There has been a lack of extensive research comparing the effects of dexmedetomidine, when used as an adjuvant, in different doses with intrathecal ropivacaine during cesarean section. Therefore, this study aimed to analyze the efficacy of varying doses of dexmedetomidine as an additive to 0.75% hyperbaric ropivacaine. In this study, we hypothesized that the inclusion of dexmedetomidine as an adjuvant to intrathecal 0.75% hyperbaric ropivacaine during cesarean section could enhance intraoperative blockade conditions, extend analgesic duration in the post-operation period, and maintain minimal impact on motor block while presenting negligible side effects.

In this randomized trial, our primary aim was to evaluate the effects of 5 µg, 7.5 µg, and 10 µg doses of dexmedetomidine added to hyperbaric 0.75% ropivacaine on the duration of analgesia in parturients scheduled for cesarean section. Furthermore, the onset of sensory and motor block, hemodynamics, sedation, and adverse effects were investigated.

## Methods

This prospective, double-blind (patient and assessor-blinded) randomized trial was conducted from November 2023 to April 2024. This research was approved by the Institutional Ethics Committee of Government Medical College, Kadapa (approval no.: ACAD./E3B/2022-2023, dated: May 27, 2023), and the trial was registered in the Clinical Trials Registry-India (register no.: CTRI/2023/10/059021; URL: https://ctri.nic.in/ Clinical trials). This study was conducted in accordance with the guidelines of the Declaration of Helsinki (2013).

One hundred twenty full-term parturients aged 21-32 years, with American Society of Anesthesiologists (ASA) physical status II, who were scheduled for lower-segment cesarean delivery with subarachnoid block were included after waiving written informed consent. Exclusion criteria comprises gestational age <36 weeks; parturients with body mass index (BMI) >35 kg m^2-1^, history of more than one previous cesarean delivery, placenta previa, ruptured membranes, intrauterine growth restriction, hypertension or pre-eclampsia, diabetes or gestational diabetes, and any contraindications to regional anaesthesia, such as bleeding disorders or local site infection.

**Figure 1:**
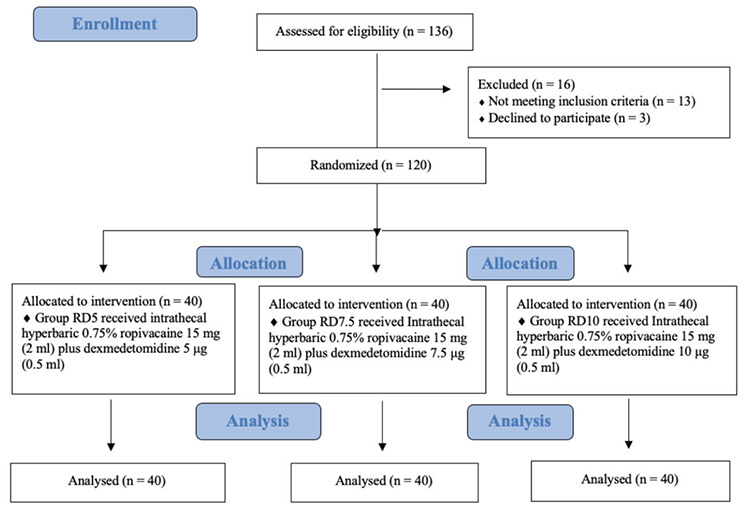
Consort diagram demonstrating the randomization.

Block randomization was performed using a computer-generated block random number table to randomly assign 120 full-term parturients into one of the three groups (Figure 1). The allocation sequence was concealed within sealed envelopes and opened by a senior resident who was not involved in the investigation. The study solutions were prepared under sterile conditions in advance and enclosed within masked 3 mL syringes according to randomization to maintain blinding. The treatment group remained unknown to the anaesthesiologist monitoring the patient, collected data, and administered the block. The three groups were designated as follows: group RD5, which received intrathecal 0.75% hyperbaric ropivacaine 2 mL plus dexmedetomidine 5 µg (diluted in 0.5 mL normal saline); group RD7.5, which received intrathecal 0.75% hyperbaric ropivacaine 2 mL plus dexmedetomidine 7.5 µg (diluted in 0.5 mL normal saline); and group RD10, which received intrathecal 0.75% hyperbaric ropivacaine 2 mL plus dexmedetomidine 10 µg (diluted in 0.5 mL normal saline).

As per institutional protocol, all patients were given 150 mg of oral ranitidine the night before surgery and were usually fasted prior to surgery. Standardized monitoring, including oxygen saturation (SpO2), pulse rate, systolic and diastolic non-invasive blood pressure, and electrocardiogram measurements, was performed during the perioperative phase. Intravenous access was established using an 18/20-G cannula, with all patients receiving a preload of 10 mL kg^-1^ of crystalloid solution. Pantoprazole 40 mg and ondansetron 4 mg were administered intravenously. Spinal anaesthesia was administered in the sitting position using a 26-G Quinke needle under strict aseptic and antiseptic precautions in the L3-L4 intervertebral space. All patients received 2.5 mL of the drug, regardless of their study group. After intrathecal injection, patients were positioned supine, and their vital signs [heart rate, systolic blood pressure (SBP), DBP, and SpO2] were recorded at 2, 5, 10, and 15 min intervals until the surgery concluded, followed by every 30 min for 6 hours during the postoperative period. A SBP below 90 mmHg or a drop of more than 20% from the basal systolic pressure was referred to as hypotension. Hypotension was managed with a bolus of 100 µg of i.v phenylephrine, and repeated if necessary. Bradycardia, which was defined as a heart rate below 60 beats per minute, was managed with i.v atropine (0.6 mg).

An 18-G epidural needle was gently inserted along the medioventral line to assess the degree of sensory block loss due to pinprick. The onset time was defined as the duration from drug administration into the subarachnoid space until the achievement of the T10 sensory block level. The length of the sensory block was defined as the time interval between the onset and two-segment regression of the block. The lower limb motor block level was determined using the Modified Bromage Score.^10^

The motor block onset time was defined as the time interval between spinal drug administration and the achievement of a Modified Bromage Score of 1. The length of spinal analgesia was defined as the time interval from intrathecal injection to the first time the patient required postoperative analgesia. Analgesic effectiveness was assessed by visual analogue scale (VAS), ranging from 0 to 10 cm scores (0=no pain, 10=most severe pain) recorded on marked paper strips intraoperatively every 15 minutes and postoperatively every half-hour until the first rescue analgesic was administered. Rescue analgesia (1 gm i.v paracetamol) was administered if the VAS score exceeded 3. Adverse events, like sedation, postoperative nausea and vomiting were recorded and treated accordingly. Sedation levels were evaluated using the modified Ramsay sedation scale.^11^

### Statistical Analysis

Power analysis was conducted based on the findings of a prior study by Kapinegowda et al.,^12^ which demonstrated that dexmedetomidine can decrease the onset time of sensory and motor blockade while prolonging the duration of anaesthesia and analgesia in a dose‑proportional fashion. Based on these results, with 5% type 1 error and 80% power, a minimum sample size of 37 patients per group was required. To validate the results, we included 40 patients in each group. Using Statistical Package for the Social Sciences (SPSS, IBM, Armonk, NY, USA) version 23.0 for Windows, data was analyzed. Continuous and categorical variables are represented as mean [standard deviation (SD)] and frequencies (percentages), respectively. To determine the association between quantitative continuous variables, one-way ANOVA followed by Bonferroni’s multiple comparison test was used. To assess the association between qualitative variables, the chi-square test followed by pairwise comparison was used. *P* value of <0.05 was considered statistically significant.

## Results

One hundred and thirty-six patients were evaluated for acceptability, and 120 patients were equally distributed among the study groups through randomization. Sixteen patients were not included in the randomization process because they either declined to provide consent or did not meet all eligibility criteria. Figure 1 illustrates the patient flow in the investigation according to Consolidated Standards of Reporting Trials recommendations.

Age, height, weight, BMI, gestational age, fetal delivery duration, and surgery duration were comparable between the three groups (Table 1). No statistically significant differences were observed in baseline hemodynamic variables among the three study groups (*P* > 0.05).

The onset of sensory and motor blocks was significantly quicker in group RD10 than in groups RD7.5 and RD5. There was a dose-related significant curtailment of the mean time to the highest sensory block (mean ± SD, 2.96±1.32, 2.26±1.50, and 1.96±0.93 min; *P* < 0.001) and mean time to the highest motor block (5.96±0.72, 5.60±1.16 and 5.43±1.075 min; *P *< 0.001) with increasing dexmedetomidine doses of 5, 7.5, and 10 µg, respectively. The time required to reach the highest level of sensory block was statistically insignificant across the groups (*P*=0.402). However, the time taken for two-segment sensory regression was significantly different among the groups: 97.26±33.67 min in group RD5, 119.18±34.27 min in group RD7.5, and 127.46±31.24 min in group RD10 (*P*=0.014). This difference indicates an earlier regression in group RD5 compared with groups RD7.5 and RD10 (Group RD5 < Group RD7.5 < Group RD10) (Table 2).

The sensory reclamation time was greatest in group RD10 compared with groups RD7.5 and RD5 (RD10 > RD7.5 > RD5). The times required for total sensory reclamation (Table 2) was 328.83±63.41 min in RD5, 345.13±66.38 min in RD7.5, and 421.21±94 min in RD10 which is statistically highly significant (*P *≤ 0.001).

Motor blockade onset was observed in RD10 at 6.40±0.14 min, RD7.5 at 8.63±0.58 min, and RD5 at 9.63±0.11 min. Motor block onset was earlier in RD10 than in both RD7.5 and RD5 (RD10 < RD7.5 < RD5), which was statistically significant (*P* ≤ 0.001).

The total motor blockade duration in RD10 was a comparatively prolonged duration of 411.23±84.41 min, than RD7.5 (364.23±82 min) and in RD5 was (331.93±83.67 min), which was statistically significant (*P*=0.046).

Similarly, the total duration of analgesia was significantly longer in RD10 (537.86±73.30 min) than in RD7.5 (451.68±64.11 min) and RD5 (417.42±68.05 min), which was statistically significant (*P *≤ 0.001). The findings indicated that the dosage had a direct impact on the total duration of motor blockade; the more the dosage, the more prolonged the block.

Adverse effects, such as sedation, bradycardia, and vomiting, were higher in patients with RD10 than in those with RD7.5 and RD5 (Table 3). However, regarding the incidence of hypotension, no significant difference was observed between the groups (*P*=0.364). Bradycardia was well managed with a solitary dose of 0.6 mg atropine sulfate i.v and did not recur. Without further deterioration, hypotension was managed with a 200 mL bolus of isotonic i.v fluids and a bolus of 100 µg of i.v phenylephrine, and repeated if necessary. Notably, a significantly greater number of patients in group RD10 exhibited a maximum sedation score (>3) than those in groups RD7.5 and RD5 (*P*=0.044).

There were no significant differences in neonatal Apgar scores or analyses of umbilical cord blood gas, which included pH, partial pressure of oxygen, and partial pressure of carbon dioxide among the groups (*P* > 0.05) (Table 4).

## Discussion

**Figure 2:**
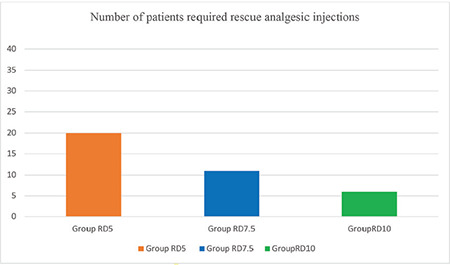
Rescue analgesic demand in postoperative period (*P*=0.037).

In this randomized prospective research, it was observed that administering 5 µg, 7.5 µg and 10 µg of dexmedetomidine with 0.75% hyperbaric ropivacaine for spinal anaesthesia in parturients scheduled for elective lower uterine segment cesarean section accelerated the onset of sensory and motor blockade, prolonged the duration, and enhanced postoperative analgesia in a dose-dependent manner. A comparatively smaller number of patients (15%) in group RD10 required i.v paracetamol [1 gm i.v] as a rescue analgesic than those from group RD7.5 (27.5%) and group RD5 (50%) (Figure 2). Bradycardia incidence was elevated in the RLD10 group (47.5%) compared with the RD7.5 and RD5 groups. Higher dexmedetomidine doses also led to increased sedation (*P*=0.044).

Dexmedetomidine is a centrally acting α2 adrenergic agonist that is eight times more selective than clonidine. It is a safe and useful adjuvant in a variety of anaesthetic and analgesic protocols. It has sedative, sympatholytic, and analgesic effects.^13^ It does not include stabilizers or additives and is offered as a solution without preservatives. It prolongs motor and sensory blockade by local anaesthetics when administered intrathecally as an adjuvant, thereby providing supraspinal analgesia. This could be a consequence of the synergistic or cumulative effect of various modes of action of different local anaesthetics. The principal focus of this study was to evaluate the potency and safety of dexmedetomidine at three different doses when combined with 0.75% hyperbaric ropivacaine in achieving adequate intraoperative anaesthesia and elongation of analgesia duration during spinal anaesthesia.

In several experimental and clinical studies, dexmedetomidine was successfully used in neuraxial blocks without inducing neurological deficits. Its intrathecal administration in humans has been advocated for concentrations ranging from 2.5 µg to 15 µg in conjunction with numerous local anaesthetics.

In this study, the onset of sensory and motor blockade was observed to occur sooner with a high dose of dexmedetomidine (10 µg) than with dexmedetomidine doses of 7.5 µg and 5 µg. These findings align with those of Halvadia and Patel,^14^ who reported that subarachnoid administration of dexmedetomidine, in conjunction with hyperbaric 0.5% bupivacaine, accelerated the onset of sensory and motor block onset. In the present prospective study, we also observed a significant and consistent lengthening of the duration of sensory and motor block with increasing subarachnoid dexmedetomidine dosage. Sudheesh et al.^15^ also reported a similar finding, where the authors compared doses of 3 µg and 5 µg of dexmedetomidine combined with 0.5% bupivacaine (4 mg) in 50 patients who underwent ambulatory surgeries for perianal diseases. They observed significant dose-related escalation in both sensory and motor block durations.

Another study by Modir et al.^16^ concluded that the duration of analgesia was prolonged in parturients who received a higher dose of dexmedetomidine (7.5 µg) in 2.5 mL of heavy 0.75% ropivacaine compared with 2.5 and 5 µg of dexmedetomidine in spinal anaesthesia. They also found that the addition of 7.5 µg of dexmedetomidine to intrathecal 0.75% ropivacaine heavy produced stable haemodynamic parameters and block characteristics compared with lower intrathecal dexmedetomidine doses in patients scheduled for cesarean section. However, likely complications, such as falls in both blood pressure and heart rate, should be taken into account simultaneously. These results are similar to those of our study.

The total analgesia duration showed a dose-dependent relationship across groups RD5 (362.58±79.87 min), group RD7.5 (398.74±73.59 min), and RD10 (483.43±76.21 min), which was statistically significant (*P *≤ 0.001). Prior studies have noted significant dose-dependent differences among the groups, indicating similar findings.^17, 18^ In a comparative study by Gupta et al.,^19^ the influence of three doses of dexmedetomidine (dexmedetomidine 2.5 µg, 5 µg, and 10 µg) combined with 15 mg of bupivacaine heavy 0.5% was studied and assigned into three groups (n = 30) on patients undergoing elective lower limb and abdominal surgeries. As in previous investigations, they examined both sensory and motor blockade properties and also differential analgesia, (the differential analgesia is defined as the interval between the end of the motor blockade and the first analgesic demand).^20^ Researchers discovered that an increase in intrathecally administered dexmedetomidine dosage from 2.5 µg to 10 µg led to increases in motor block, sensory block, and analgesia durations of 41.28%, 67.28%, and 208.37%, respectively. Prolonged analgesia duration has the advantage of reducing the incidence of complications of postoperative pain (e.g., the risk of neuro-sensitization, delayed wound healing, prolonged hospitalization), thereby minimizing chronic pain and prolonged motor blockade-related issues, such as deep venous thrombosis, reduced mobilization, and pulmonary embolism.

Keplinger et al.^21^ studied the dose dependency of dexmedetomidine when combined to ropivacaine in peripheral nerve blockade. In this investigation, 22.5 mg of only ropivacaine (R) or in combination with 50 µg (RD50), 100 µg (RD100), or 150 µg (RD150) of dexmedetomidine was administered as an ulnar nerve block to each subject. A significant increase was observed in the mean duration (SD) of analgesia with dexmedetomidine when administered at increasing doses: R = 8.7 h, RD50 = 16.4 h, RD100 = 20.4 h, and RD150 = 21.2 h. Additionally, there was a dose-dependent increase in sedation. These outcomes are consistent with our results. Adding dexmedetomidine potentiated the analgesic effect of 0.75% hyperbaric ropivacaine in spinal anaesthesia in a dose-related manner. In this study, a lower number of patients in the RD10 group required i.v paracetamol as rescue analgesia compared with groups RD5 and RD7.5 (*P* < 0.05). Our observations are consistent with those of Bi et al.^22^. Similarly, Kapinegowda et al.^12^ also observed that i.v diclofenac sodium (aqueous base) 75 mg was administered as a rescue analgesic in the subarachnoid 0.5% heavy bupivacaine combined with the 10 µg dexmedetomidine group compared to 7.5 µg and 5 µg dexmedetomidine groups for infra umbilical surgeries.^13^

In this study, adverse effects, especially sedation, bradycardia, and vomiting, were exhibited at a notably higher frequency in group RD10 than in groups RD5 and RD7.5. The occurrence of hypotension did not exhibit statistical significance across the three groups. These adverse reactions are typically manageable. Dexmedetomidine’s sedative characteristics of dexmedetomidine stem from its lipophilic properties, leading to systemic absorption upon intrathecal administration. Significant sedation was observed with larger doses of dexmedetomidine (1.5 µg kg^-1^) in conjunction with caudal ropivacaine compared with plain ropivacaine for postoperative analgesia in pediatric ambulatory surgeries.^23^ However, this did not result in patient discharge delays.

### Study Limitations

We only included healthy individuals with ASA II in our study. The effects of intrathecal in patients with ASA III and IV and those with comorbidities have not yet been studied. Another limitation was that participants with a BMI >35 kg m^-2^ and age >32 years were not included in our study. Therefore, the findings cannot be applied to pregnant women. Furthermore, a lengthy postoperative follow-up was lacking in our investigation to identify possible neurological problems. Despite these limitations, it is important to highlight that this study produced several important findings. Additionally, prospective studies are required to determine the efficacy of various dexmedetomidine doses as adjuvants in neuraxial block for parturients undergoing lower segment cesarean section.

## Conclusion

Adding 10 µg of dexmedetomidine to hyperbaric 0.75% ropivacaine in spinal anaesthesia results in a prolonged analgesic effect compared with doses of 7.5 µg and 5 µg. Additionally, the higher dosage enhances the onset and extends the duration of sensorimotor blockade. However, higher dexmedetomidine doses result in a higher incidence of sedation and bradycardia, necessitating close monitoring.

## Ethics

**Ethics Committee Approval:** This research was approved by the Institutional Ethics Committee of Government Medical College, Kadapa (approval no.: ACAD./E3B/2022-2023, dated: May 27, 2023).

**Informed Consent:** Written informed consent was obtained from all participants.

## Figures and Tables

**Table 1. Demographic Data:** 

**Variable**	**RD5 (n = 40)**	**RD7.5 (n = 40)**	**RD10 (n = 40)**	***P* value**
Age (years)	26.9±5.1	25.2±6.4	25.8±4.7	0.552
BMI (kg m^-2^)	23.04±2.50	23.05±2.81	22.91±2.74	0.663
Gestational week (weeks)	38.6±1.1	38.2±1.4	37.8±1.9	0.069
Fetal delivery time (min)	23.7±5.2	24.0±3.7	23.6±4.7	0.124
Duration of surgery (min)	57.2±10.3	58.0±9.1	60.4±13.9	0.194

Data are expressed as mean ± SD.

SD, standard deviation; BMI, body mass index

**Table 2. Properties of Subarachnoid Blocks:** 

**Variable**	**RD5 (n = 40)**	**RD7.5 (n = 40)**	**RD10 (n = 40)**	***P* value**
Time of onset of sensory block (min)	2.96±1.32	2.26±1.50	1.96±0.93	0.025
Time to achieve maximum Level of the sensory block (min)	5.96±0.72	5.60±1.16	5.43±1.075	0.402
TTSSR (min)	97.26±33.67	119.18±34.27	127.46±31.24	0.014
TCSR time to complete Sensory recovery (min)	328.83±63.41	345.13±66.38	421.21±94.6	<0.001
Total duration analgesia (min)	362.58±79.87	398.74±73.59	483.43±76.21	<0.001
Time to rescue analgesia (min)	417.42±68.05	451.68±64.11	537.86±73.30	<0.001
Time of onset of motor blockade (min)	9.63±0.11	8.63±0.58	6.40±0.14	<0.001
Total duration of motor blockade (min)	331.93±83.67	364.23±82.39	411.23±84.41	0.046

TCSR, time to complete sensory recovery; TTSSR, time taken for two-segment sensory regression

**Table 3. Comparison of Adverse Events:** 

**Side effect**	**Group RD5, n (%)**	**Group RD7.5, n (%)**	**Group RD10, n (%)**	***P* value**
Sedation score (>3)	11 (27.5%)	15 (37.5%)	18 (45%)	0.044
Bradycardia (HR <50 bpm)	6 (15%)	12 (30%)	19 (47.5%)	0.020
Hypotension (MAP <60 mmHg)	5 (12.5%)	5 (12.5%)	12 (30%)	0.364
Vomiting	0	0	2 (5%)	0.024

Values are illustrated as number of patients.

HR, heart rate; MAP, mean arterial pressure

**Table 4. Neonatal Umbilical Cord Blood Air and Apgar Scores:** 

**Parameter**	**Group RD5**	**Group RD7.5**	**Group RD10**	***P* value**
PH	7.34 (7.32, 7.37)	7.36 (7.33, 7.38)	7.34 (7.32, 7.37)	0.464
PO_2_	30.0 (22.5, 35.0)	32.0 (26.0, 39.0)	31.0 (21.0, 37.5)	0.491
PCO_2_	43.0 (39.0, 46.5)	46.0 (39.0, 48.5)	43.0 (39.0, 47.0)	0.820
1 min Apgar	9.0 (8.0, 9.0)	8.5 (8.0, 9.0)	9.0 (8.0, 9.0)	0.061
5 min Apgar	10.0 (10.0, 10.0)	10.0 (10.0, 10.0)	9.0 (9.0, 10.0)	0.368

Data are expressed as medians
